# A low psoas muscle volume correlates with a longer hospitalization after radical cystectomy

**DOI:** 10.1186/s12894-017-0279-2

**Published:** 2017-09-18

**Authors:** Yoko Saitoh-Maeda, Takashi Kawahara, Yasuhide Miyoshi, Sohgo Tsutsumi, Daiji Takamoto, Kota Shimokihara, Yuutaro Hayashi, Taku Mochizuki, Mari Ohtaka, Manami Nakamura, Yusuke Hattori, Jun-ichi Teranishi, Yasushi Yumura, Kimito Osaka, Hiroki Ito, Kazuhide Makiyama, Noboru Nakaigawa, Masahiro Yao, Hiroji Uemura

**Affiliations:** 1 Departments of Urology and Renal Transplantation, Yokohama City University Medical Center, 4-57 Urafune-cho, Minami-ku, Yokohama, Kanagawa 2320024 Japan; 2 Department of Urology, Yokohama City University Graduate School of Medicine, Yokohama, Japan

**Keywords:** Bladder cancer, Radical cystectomy, Sarcopenia, Psoas muscle

## Abstract

**Background:**

Recently, sarcopenia has been reported as a new predictor for patient outcomes or likelihood of post-operative complications. The purpose of this study was to evaluate the association of the psoas muscle volume with the length of hospitalization among patients undergoing radical cystectomy.

**Methods:**

A total of 63 (80.8%) male patients and 15 (19.2%) female patients who underwent radical cystectomy for their bladder cancer in our institution from 2000 to 2015 were analyzed. The psoas muscle index (PMI) was calculated by normalizing the psoas muscle area calculated using axial computed tomography at the level of the umbilicus (cm^2^) by the square of the body height (m^2^). Longer hospitalization was defined as hospitalization exceeding 30 days after surgery.

**Results:**

The median PMIs (mean ± standard deviation) were 391 (394 ± 92.1) and 271 (278 ± 92.6) cm^2^/m^2^ in men and women, respectively. Thus, the PMIs of male patients were significantly larger than those of females (*p* < 0.001). Based on the differences in gender, we analyzed 63 male patients for a further analysis. In male patients, those hospitalized longer showed a significantly smaller PMI than those normally discharged (377 ± 93.1 vs. 425 ± 83.4; *p* = 0.04). Similarly, male patients with a small PMI (<400) had a significantly worse overall survival (*p* = 0.02) than those with a large PMI (≥400).

**Conclusions:**

The presence of sarcopenia was found to be associated with significantly longer hospitalization after radical cystectomy in male patients. Furthermore, in men, a PMI <400 may suggest a significantly worse prognosis.

## Background

For locally advanced bladder cancer, radical cystectomy is still the gold standard therapy [1, 2]. However, despite its effectiveness, the perioperative complication rate is reported to be around 30%, and the 30- and 90-day post-operative mortality rates are 3.2% and 5.2%, respectively [1–3]. The indication for radical cystectomy is usually considered based on the patient’s age, complications, and performance status [4, 5]. Recently, sarcopenia was reported as a new predictor for the prognosis or risk of post-operative complications [1, 6, 7].

Sarcopenia is the age-related loss of skeletal muscle mass [8]. Previous studies have defined the sum of the muscle masses of the four limbs as the appendicular skeletal mass in order to calculate the psoas muscle index (PMI) [9, 10]. A correlation between sarcopenia and oncologic outcomes has been reported in malignant melanoma, breast cancer, and hepatocellular carcinoma [11–14]. In patients with bladder cancer, several studies have suggested that sarcopenia correlates with a worse prognosis than in those without sarcopenia [1, 7]. However, whether or not the PMI easily determined using the one-side psoas volume in non-contrast computed tomography (CT) precisely predicts post-operative complications as well as the long-term oncologic outcomes in patients undergoing radical cystectomy remains controversial.

We therefore explored the value of sarcopenia in bladder cancer patients who underwent radical cystectomy.

## Methods

### Patients

A total of 78 patients (63 males and 15 females) underwent radical cystectomy for bladder cancer at Yokohama City University Medical Center (Yokohama, Japan) from 2000 to 2015. All of the patients were Japanese. The institutional review board of Yokohama City University Medical Center approved this study [*D1507018*]. The patients were followed up every three months for two years after cystectomy and every six months thereafter using CT.

### Clinical assessments

The volume and area of the psoas muscle were calculated using axial CT at the level of the umbilicus before radical cystectomy. The PMI (cm^2^/m^2^) was calculated by normalizing the psoas muscle area (cm^2^) by the square of the body height (m^2^).

Longer hospitalization was defined as hospitalization exceeding 30 days after surgery. Based on observed differences in gender, we analyzed the 63 male patients in a further analysis. The overall survival (OS) was compared between the high- (≥400) and low- (<400) PMI groups. The patients’ perioperative complications were assessed and scored according to the modified Clavien grading system.

### Statistical analysis

The patients’ characteristics and preoperative factors were analyzed using the Mann-Whitney *U* and chi-squared tests. The Kaplan-Meier product limit estimator was used to estimate the OS. The survival duration was defined as the time between radical cystectomy and death. The log-rank test was performed for comparison. A *p* value of <0.05 was considered to be statistically significant.

## Results

### Patients’ characteristics

The median/mean (± standard deviation (SD)) follow-up times in male and female patients after radical cystectomy were 24.8/36.6 (± 30.9) and 25.4/31.5 (± 24.6) months, respectively The median/mean (± SD) durations of post-operative hospitalization were 36/39.7 (± 17.4) days in male patients and 37.0/42.1 (± 15.3) days in female patients.

### Length of hospitalization vs. psoas muscle volume

**Fig. 1:**
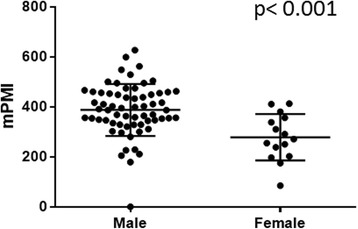
PMI in male versus female bladder cancer patients undergoing radical cystectomy

**Fig. 2:**
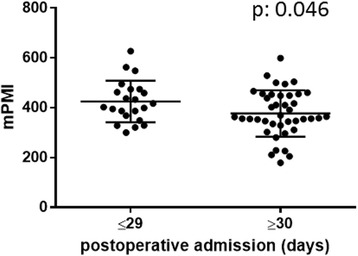
PMI and duration of postoperative admission in male patients

The median/mean (± SD) psoas areas of the 63 male and 15 female patients were 1078/1085 (± 254) and 632/634 (± 239) cm^2^, respectively, and the PMIs were 391/393 (± 92.1) and 271/278 (± 92.6) cm^2^/m^2^, respectively. Thus, male patients had a significantly higher PMI than female patients (*p* < 0.001, Fig. 1). Among the male patients, those hospitalized longer showed a significantly smaller psoas muscle volume than those normally discharged (Fig. 2). A similar trend was noted among female patients (longer hospitalized group: 763/1045 ± 826 cm^2^/m^2^ vs. control group: 774/670 ± 373 cm^2^/m^2^), but the difference was not statistically significant (*p* = 0.405), possibly due to the small number of female subjects.

### Histopathological features

Histopathological features in male patients, including tumor grade, pathological T stage, lymph node metastasis, and the presence of concurrent carcinoma in situ (CIS), are summarized in Table 1. The frequencies of clinical T and N stage showed no marked differences between the high- and low-PMI groups. In the male, older patients tended to have a lower PMI than the younger patients; however, the difference did not reach statistical significance. The correlation coefficient (R2) was 0.022.

**Table 1:** Patient characteristics and psoas muscle volume in male patients

	Total (male only)	PMI < 400	PMI ≥ 400	*P* value
	(*n* = 63)	(*n* = 34)	(*n* = 29)	
Age				
< 65 years	30 (47.6%)	12 (35.3%)	18 (62.1%)	0.062
≥ 65 years	33 (52.4%)	22 (64.7%)	11 (37.9%)	
Pathological Tumor Grade				
1	1 (1.2%)	0 (0%)	1 (38.5%)	0.477
2	25 (39.7%)	13 (41.0%)	12 (46.1%)	
3	32 (50%)	19 (59.4%)	13 (50%)	
Pathological T Stage				
≤ pT2	46 (74.2%)	21 (63.6%)	25 (93.1%)	0.047
≥ pT3	14 (25.8%)	12 (37.4%)	2 (6.9%)	
Lymph Node Metastasis				
pN0	56 (88.9%)	30 (88.2%)	26 (89.7%)	0.097
pN+	7 (11.1%)	4 (11.8%)	3 (10.3%)	
Concurrent CIS				
Yes	12 (19.0%)	5 (14.7%)	7 (24.1%)	0.521
No	51 (81.0%)	29 (85.3%)	22 (75.9%)	
Prognosis				
Death	10 (15.9%)	8 (23.5%)	2 (6.9%)	0.258
Alive	53 (84.1%)	26 (76.5%)	27 (93.1%)	
Body Height				
Median, Mean ± SD	166, 166 ± 5.84	166, 165 ± 6.32	167, 167 ± 5.31	0.595
Psoas area (cm2)				
Median, Mean ± SD	1078, 1085 ± 255	920, 896 ± 152	1297,1307 ± 149	<0.001
Psoas Muscle Index (cm2/m2)				
Median, Mean ± SD	391, 393 ± 92.1	347,327 ± 57.3	347, 472 ± 56.7	<0.001

### Correlation of PMI with the OS

**Fig. 3:**
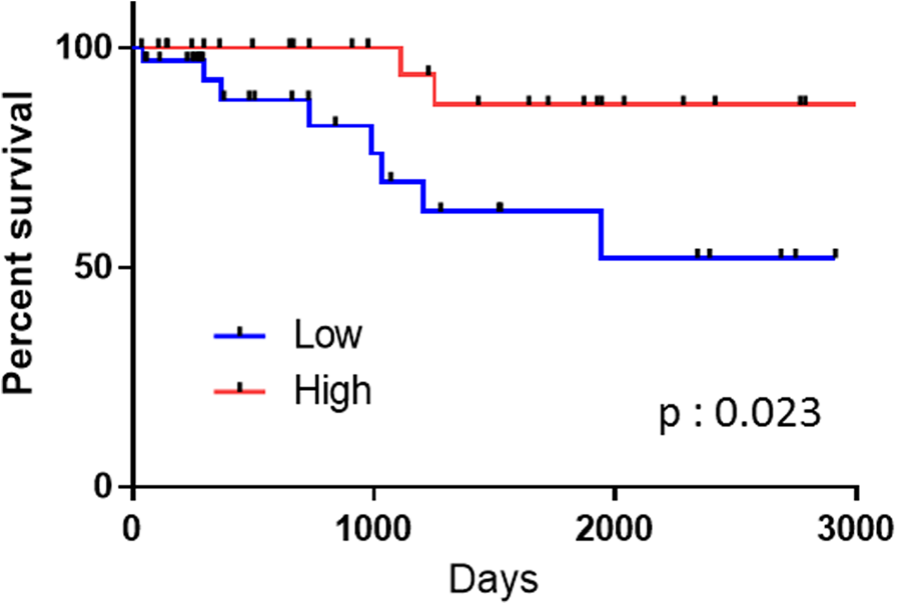
Overall survival in male patients with high and low PMIs

The OS was compared in male patients with high versus low PMI. Kaplan-Meier and log-rank tests revealed that the patients with a high PMI had a significantly better OS than those with a low PMI (*p* = 0.023, Fig. 3). The mean survivals were 2889 days in the high-PMI group and 2009 days in the low-PMI group.

### Perioperative complications

In male patients, the low-PMI group showed a significantly higher rate of complications than the high-PMI group (82.9% vs 31.8%, *p* < 0.001). Furthermore, the patients in the low-PMI group experienced severe complications (Clavien grade ≥ 3, 19.5%) (Table 2).

**Table 2:** Postoperative complications (male patients only)

Clavien Grading Score	PMI < 400	PMI ≥ 400	*p* value
0	7 (17.1%)	15 (68.2%)	<0.001
1	24 (58.5%)	6 (27.3%)	
2	2 (4.9%)	1 (4.5%)	
≥3	8 (19.5%)	0 (0.0%)	

## Discussion

Sarcopenia is defined as a low volume of skeletal muscle. Sarcopenic patients show a worse swallowing function and nutritional condition than those without sarcopenia [15]. One study reported that sarcopenic patients had a lower activity of daily life than those without sarcopenia at ≥65 years of age [16]. Recently, sarcopenia has been reported as a predictive factor for postoperative complications and the survival in several cancers. For instance, in patients with stage 2 or 3 gastric cancer undergoing gastrectomy, sarcopenia was found to be correlated with higher rates of postoperative complications and a poorer overall and disease-free survival than in those without sarcopenia [17]. Another study found that male sarcopenia patients who underwent pancreatectomy showed a poorer overall survival than those without sarcopenia [18].

Although the detailed mechanism underlying the association between sarcopenia and post-operative complications remains unknown, body frailty is suspected to be involved, as body failure or reduced body durability results in longer admission duration [19]. Sarcopenia develops due to body frailty with aging or in the presence of malignant disease.

The present study showed that, in male patients, those with a lower psoas muscle volume who underwent radical cystectomy had a longer hospitalization than those with a normal volume. In bladder cancer, there have been several studies regarding sarcopenia in patients undergoing radical cystectomy. Psutka et al. reported that, compared with non-sarcopenic patients, sarcopenic patients showed a significantly lower cancer-specific 5-year survival (49% vs 72%; *p* = 0.003) and OS (39% vs. 70%; *p* = 0.003) [1]. Wan et al. showed that sarcopenia increased the risk of severe complications after radical cystectomy [20]. Smith et al. reported that sarcopenic female patients had an increased risk of post-operative complications compared with non-sarcopenic patients [21]. Consistent with these data, our results showed that a lower preoperative psoas muscle volume was associated with a prolonged hospitalization after radical cystectomy, suggesting that the psoas muscle volume might be a reliable factor for predicting a long hospitalization, presumably due to postoperative complications.

Most patients who undergo radical cystectomy for muscle-invasive bladder cancer are relatively old. Accordingly, predicting postoperative complications is important before performing radical cystectomy in such vulnerable patients. Thus far, combination therapy, including transurethral resection, systemic chemotherapy, and radiation therapy, have been thought to be the most effective bladder-preserving therapies, with a 5-year survival rate of around 50% to 60% [2, 22]. In patients over 70 years of age, intra-arterial systemic chemotherapy combined with radiation has been shown to be associated with a more favorable prognosis than radical cystectomy. Similarly, if radical cystectomy is contraindicated due to advanced age, sarcopenic patients may also be good candidates for bladder preservation.

According to the previous studies on the association of sarcopenia with the prognosis or postoperative complications, dual-energy X-ray absorptiometry and bioelectrical impedance analysis have been used to detect muscle volume. We used standard axial CT at the level of the umbilicus. A low psoas muscle volume detected by CT in this manner was associated with a longer post-operative admission due to postoperative complications. Our method is easy to perform, and in most patients undergoing radical cystectomy, no additional procedures for measuring the psoas muscle volume are required.

## Conclusion

In the present study, we showed that sarcopenia is a predictor of longer hospitalization, and sarcopenic patients had a significantly worse OS than those without sarcopenia among male patients. The present findings support sarcopenia as a meaningful factor influencing the choice of therapy for locally advanced bladder cancer.

## Abbreviations

OS: Overall survival
PMI: Psoas muscle index
SD: Standard deviation
